# m6A Regulator-Mediated RNA Methylation Modification Patterns Regulate the Immune Microenvironment in Osteoarthritis

**DOI:** 10.3389/fgene.2022.921256

**Published:** 2022-06-23

**Authors:** Yang Duan, Cheng Yu, Meiping Yan, Yuzhen Ouyang, Songjia Ni

**Affiliations:** ^1^ Department of Spinal Surgery, Zhujiang Hospital, Southern Medical University, Guangdong, China; ^2^ Outpatient Department, Zhujiang Hospital, Southern Medical University, Guangdong, China; ^3^ Air Force Hospital of Southern Theater Command of the People’s Liberation Army, Guangdong, China; ^4^ Department of Orthopedics and Traumatology, Zhujiang Hospital, Southern Medical University, Guangdong, China

**Keywords:** epigenetics, immune microenvironment, RNA n6 methyl adenosine, osteoarthritis, RNA modification

## Abstract

Epigenetic regulation, particularly RNA n6 methyl adenosine (m6A) modification, plays an important role in the immune response. However, the regulatory role of m6A in the immune microenvironment in osteoarthritis (OA) remains unclear. Accordingly, we systematically studied RNA modification patterns mediated by 23 m6A regulators in 38 samples and discussed the characteristics of the immune microenvironment modified by m6A. Next, we constructed a novel OA m6A nomogram, an m6A-transcription factor-miRNA network, and a drug network. Healthy and OA samples showed distinct m6A regulatory factor expression patterns. *YTHDF3* expression was upregulated in OA samples and positively correlated with type II helper cells and *TGFb* family member receptors. Furthermore, three different RNA modification patterns were mediated by 23 m6A regulatory factors; in Mode 3, the expression levels of *YTHDF3*, type II T helper cells, and *TGFb* family member receptors were upregulated. Pathways related to endoplasmic reticulum oxidative stress and mitochondrial autophagy showed a strong correlation with the regulatory factors associated with Mode 3 and 23 m6A regulatory factors. Through RT-qPCR we validated that *SREBF2* and *EGR1* as transcription factors of *YTHDF3* and *IGF2BP3* are closely associated with the development of OA, hsa-miR-340 as a miRNA for *YTHDF3* and *IGF2BP3* was involved in the development of OA, we also detected the protein expression levels of *IGF2BP3*, *YTHDF3*, *EGR1* and *SREBF2* by western blotting, and the results were consistent with PCR. Overall, the constructed nomogram can facilitate the prediction of OA risk.

## Introduction

Osteoarthritis (OA) is a degenerative multifactorial disease that is characterized by progressive joint failure, which is often associated with joint pain, stiffness, and decreased range of motion (Lopes et al., 2017; Endisha et al., 2021). The pathological changes associated with OA include cartilage degeneration, synovitis, fibrosis, and subchondral bone sclerosis. The etiology of OA is complex and is currently thought to result from a combination of biomechanical processes, trauma, chronic inflammation, and immune response. Previous studies have shown that a variety of cells, cytokines, chemokines, complements, and other immune system factors are involved in OA pathogenesis (Kandahari et al., 2015). However, studies on the role of m6A regulatory factors in the immune regulation of OA are scarce. Thus, further studies are needed in this regard.

N6 adenosine methylation (M6A), which is the most frequently observed RNA modification type, extensively occurs in mRNA, lncRNAs, and miRNAs. Specifically, M6A plays a crucial role in various physiological processes as well as in disease progression. Its modification is also a dynamic and reversible process controlled by different types of regulatory proteins, namely: methyltransferase (“writer”), demethylase (“erasers”), and the binding protein (“reader”). Furthermore, m6A modification is greatly affected by the expression and function of these regulatory proteins, and studying these regulatory proteins can enhance understanding of the role of m6A in gene regulation (Chong et al., 2021). It has also been observed that m6A modification under the influence of regulatory factors is associated with inflammation, the tumor microenvironment (TME), and immune response (Huang et al., 2021).

Previous studies have shown that m6A regulatory factors, especially *METTL3* and *FTO*, are involved in OA progression via the regulation of inflammatory response and extracellular matrix degradation. On the one hand, FTO-dependent m6A demethylation mediates the upregulation of *AC008*, which inhibits chondrocyte viability and promotes chondrocyte apoptosis and ECM degradation in OA (Yang et al., 2021). On the other hand, *METTL3* affects the stability of autophagy-related 7 (*ATG7*) mRNA, thereby influencing autophagic activity in an m6A- YTHDF2-dependent manner. This in turn promotes FLS fibroblast-like synovial cell senescence and OA progression (Chen et al., 2022). Further, *METTL3* regulates inflammatory responses in OA. It has also been observed that extracellular matrix degradation in OA is related to the balance between *TIMPs* and *MMPs*, which are regulated by *METTL3* (Sang et al., 2021). Furthermore, *METTL3* regulates cartilage tissue by regulating cartilage fine-cellular NF-kB signal transduction, while ECM synthesis plays a mediator role in OA progression (Liu et al., 2019). Moreover, there is increasing evidence that m6A is involved in the regulation of immune responses (Zhang et al., 2021). Therefore, investigating the role of m6A regulatory factors in the immune response of OA and studying the differences in immune changes between healthy and OA tissues can improve our understanding of OA pathogenesis from a completely different perspective.

In this study, we systematically evaluated the modification patterns of m6A regulatory factors in OA. Thus, we found that m6A regulatory factors were distinguished between healthy and OA samples. The abundance of OA-infiltrating immune cells and the immune response genome were found to be significantly correlated with the m6A regulator, thus suggesting the existence of a strong correlation between m6A regulators and immune regulation. We also found different immune characteristics for different m6A molecular subtypes and analyzed their biological functions. These results indicated that the m6A modification pattern has a significant effect on the immune microenvironment in OA. We also constructed an m6A transcription factor-miRNA network as well as a drug network. Simultaneously, a new m6A OA nomogram that can facilitate the prediction of OA risk was established.

## Materials and Methods

### Data Acquisition and Difference Analysis

The relevant OA dataset, GSE114007 (Fisch et al., 2018), was downloaded from the GEO database. This dataset, with data platforms GPL11154 and GPL18573, consists of sequencing data corresponding to Homo sapiens. Further, this GSE114007 dataset includes 38 samples (including 18 control samples and 20 OA samples), all of which were included in this study. The R package DEseq2 (Love et al., 2014) was used to analyze differences in m6A gene expression values between the control and OA groups. The results of the different analyses were presented as heat maps and volcanic maps using R-package heat and ggplot2, respectively.

### Analysis of m6A Regulatory Factors in OA

The expression relationships of 23 m6A regulatory factors in healthy and OA samples were evaluated using Spearman’s correlation analysis. The random forest (Ishwaran and Lu, 2019) was first used to identify m6A regulators related to OA. Thereafter, the least absolute shrinkage and selection operator (LASSO) regression (Beinse et al., 2022) was used for feature selection, dimensionality reduction, and m6A regulator classifier developments. The distinguishing performances of the signature were then evaluated via receiver operating characteristic (ROC) curve analysis.

### Analysis of the Correlation Between m6A Regulators and Immune Characteristics

Single-sample gene set enrichment analysis (ssGSEA) (Hanzelmann et al., 2013) was employed to estimate the number of specific infiltrating immune cells and the activity of specific immune responses. The absolute enrichment degree of a given gene set in each sample within a given dataset was expressed as an enrichment score. Further, gene listings for the gene set of infiltrating immune cells were obtained from previous studies (Shen et al., 2018; Zhang et al., 2020) and the immunoreaction gene set was obtained from the ImmPort database (http://www.immport.org) (Bhattacharya et al., 2014). The enrichment fraction represented the abundance of immune cells as well as the absolute enrichment of the immune response. The correlation of m6A regulators with immunocyte fractions and immune reaction activity was determined using Spearman’s correlation analysis.

### Identification of m6A Modification Pattern

Based on the expression of 23 m6A regulatory factors, different m6A modification patterns were identified via an unsupervised clustering analysis. Clustering was performed using R-package ConsonsuclusterPlus (Wilkerson and Hayes, 100). The number of clusters and robustness elation map of the patterns were also evaluated. Principal component analysis (PCA) further verified the expression patterns of the 23 m6A regulators in the different modification modes. Kruskal–Wallis test was then performed to compare the expression of m6A regulatory factors, abundance scores of infiltrating immune cells, immune response scores, and HLA gene expression levels in the three different modification modes.

### Biological Enrichment Analysis for Distinct m6A Modification Patterns

Biological signaling pathways may also reflect biological changes. Thus, we obtained the gene sets corresponding to “h.all.v7.0. symbols,” and “c5. go.v7.0. symbols” in the MSigDB database and converted the expression matrix into a pathway activation score matrix using the GSVA package. Thereafter, the activation scores of the two groups were compared using the R-package limma (Ritchie et al., 2015). Simultaneously, we also analyzed the correlation between the activation scores corresponding to the endoplasmic reticulum oxidative stress and mitochondrial autophagy pathways and the expression levels of m6A regulatory factors.

### Transcription Factors: miRNA Networks and Drug-Compound Networks

We analyzed the targeted transcription factors, targeted miRNAs, and interacting drugs corresponding to high-expression genes in OA using the NetworkAnalyst database (Zhou et al., 2019), and thereafter constructed a network diagram.

### Cartilage Donors

Normal human knee cartilage tissues were procured by tissue banks (approved by Scripps Institutional Review Board) from 5 females and 13 males (age 18–61, mean 38) without history of joint disease or trauma and processed within 24–48 h post mortem. Full thickness cartilage was harvested for RNA isolation from identical locations on the weightbearing regions on medial and lateral femoral condyles, and adjacent tissue sections were harvested for histology to verify the cartilage integrity. OA-affected cartilage was harvested from the tissue removed during knee replacement surgery from 12 female and 8 male donors (age 52–82, mean 66). Body mass indices between the normal (BMI = 32.4 ± 8.0) and OA (BMI = 30.7 ± 8.1) were not significantly (*p* = 0.506) different.

### Tissue Processing, RNA and DNA Isolation

Cartilage was stored at −20°C in Allprotect Tissue Reagent (Qiagen, V alencia, CA) immediately after harvest until RNA extraction. For RNA isolation, a minimum of 150 mg of cartilage (dry weight) was pulverized using a 6770 Freezer/Mill Cryogenic Grinder (SPEX SamplePrep, Metuchen, NJ), and homogenized in Qiazol Lysis Reagent (Qiagen, V alencia, CA) at a concentration of 25 mg tissue sample per 700 µl Qiazol. To remove proteins and cellular debris, a initial phenol-chloroform extraction was performed. Briefly, samples were mixed with 0.2 volumes of chloroform, incubated for 5 min in ice, and centrifuged a t 14,000 rpm for 15 min at 4°C. The aqueous phase was collected, mixed with 1 volume of Qiazol and incubated for 30 min in ice. Then, samples were mixed with 1 volume of 100% ethanol, loaded into a mRNeasy Mini kit column (Qiagen) and digested on-column with DNAse following manufacturer instructions. RNA was eluted in 15 µl of RNase-free water. RNA purity was assessed using NanoDrop (ND-1000, Thermo Scientific, Wilmington, DE) and RNA integrity number (RIN) was calculated using a 2100 Bioanalyzer (Agilent, Santa Clara, CA). Average RIN numbers were 6.08 ± 0.95.

### Library Preparation and Sequencing

RNA samples from 18 normal and 20 OA cartilage donors were sequenced using 150 ng of total RNA as input. Sequencing mRNA libraries were prepared using the Encore Complete RNA-Seq DR Multiplex System 1–8 and 9–16 (NuGen, San Carlos, CA) with 16 unique indexed adapters (L2V6DR-BC2-L2V6DR-BC16). Two lanes of an Illumina HiSeq 2000 instrument were used to generate a total of 8–30 million 100 bp reads. The Illumina Genome Analyzer Pipeline Software (Casava v1.8.2) was used to convert the original image data generated by the sequencing machine into sequence data via base calling in order to generate fastq files and to demultiplex the samples. We performed a per base sequence quality check using the software FastQC (v0.10.1) (http://www.bioinformatics.babraham.ac.uk/projects/fastqc/) prior to read mapping. Raw RNAseq reads were aligned to the human genome (hg19) using the STAR aligner. The number of reads sequenced per sample ranged from 19 to 24 million reads, which should be sufficient for gene level quantification, but only 2–12 million reads per sample mapped to protein coding genes. To account for this issue, we applied high stringency the filtering of lowly expressed genes (log counts per million >3) so that only the differential expression analysis included only genes that were expressed in high enough abundances to be confident in their relative gene expression values.

### Cell Culture

DMEM, 0.25% trypsin, and phosphate buffered saline (PBS) were equilibrated at room temperature and used for chondrocytes (Procell, Cat NO.: CP-H107, Wuhan, China) culture. Thereafter, the old medium was discarded and washed twice with PBS, which was followed by the addition of an appropriate amount of trypsin and digestion at 37°C for 1–2 min. The digestion was stopped by adding the culture medium, and the chondrocytes were then collected via blowing. The chondrocytes density was adjusted and the cells were inoculated into a 6-well plate, which was placed in an incubator at 37°C with 5% CO_2_; the culture was incubated overnight. The next day, the cell culture plate was removed and washed with PBS three times; 1 ml of PBS was added in each wash cycle, and the resulting solution was shaken gently to avoid washing out the cells. Thereafter, the cells in the degeneration group were added to the medium containing IL-1β(PEPROTECH) concentration of 40 ng/ml, while those in the control group were added to the normal medium. Culturing was then continued for 48 h.

### Reverse Transcription-Quantitative Polymerase Chain Reaction (RT-qPCR)

Total RNA of 1*106 cells was extracted using TRIzol reagent (15,596–026; Invitrogen, Carlsbad, CA, United States). A reverse transcription reaction system was constructed using the PrimeScript RT reagent kit with GDNA Eraser (# RR047A, Tokyo, Takara), and reverse transcription was performed onboard. The PCR system was constructed using the SYBR Green qPCR Mix (#D7260; Beyotime, Shanghai, China), and PCR detection was performed on a computer system. The 2-ΔΔCt method was used to calculate the relative expression levels in the samples during RT-qPCR data processing. The primers used are listed in Supplementary Table S1.

### Western Blotting

The total protein of 1*106 cells was extracted using a whole cell lysis assay (KeyGEN Biotech, Nanjing, Jiangsu Province, China). Eighty micrograms of sample protein were subjected to SDS-PAGE (KeyGEN Biotech) and transferred to PVDF membranes (Millipore). The membranes were blocked and probed with the indicated primary antibodies at 4 °C for 12 h. The membranes were then incubated with the indicated HRP-conjugated secondary antibodies at room temperature for 2 h, and the expression of the target proteins was detected by ECL (KeyGEN Biotech). The following antibodies were used: IGF2BP3 (MA5-27484, 1:1000; Thermo Fisher), YTHDF3 (PA5-107309, 1:1000, Thermo Fisher), EGR1 (MA5-15008, 1:2000; Thermo Fisher), SREBF2 (1:2000; Thermo Fisher) and GAPDH (1:1000; Beyotime Biotechnology, Shanghai, China).

### Statistical Analysis

All data calculations and statistical analyses were performed using R software (https://www.r-projec t. org/, version 4.0.2). Comparison between two groups of continuous normally distributed variables were realized by performing the independent Student’s t-test, and the difference between non-normally distributed variables was analyzed using the Mann-Whitney U test (Wilcoxon rank-sum test). All statistical *p* values were bilateral, and statistical significance was set at *p* < 0.05.

## Results

### Expression of the m6A-Related Genes in OA

**FIGURE 1:**
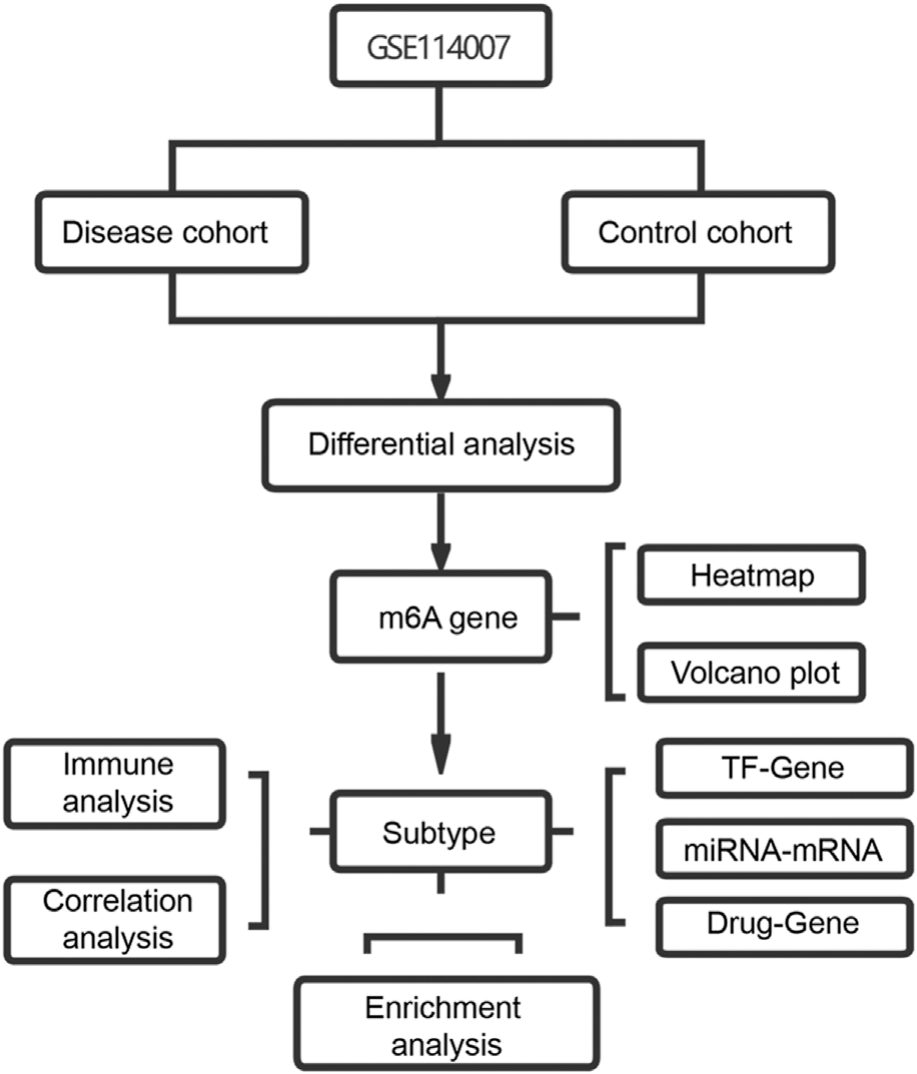
Study flow chart.

**FIGURE 2:**
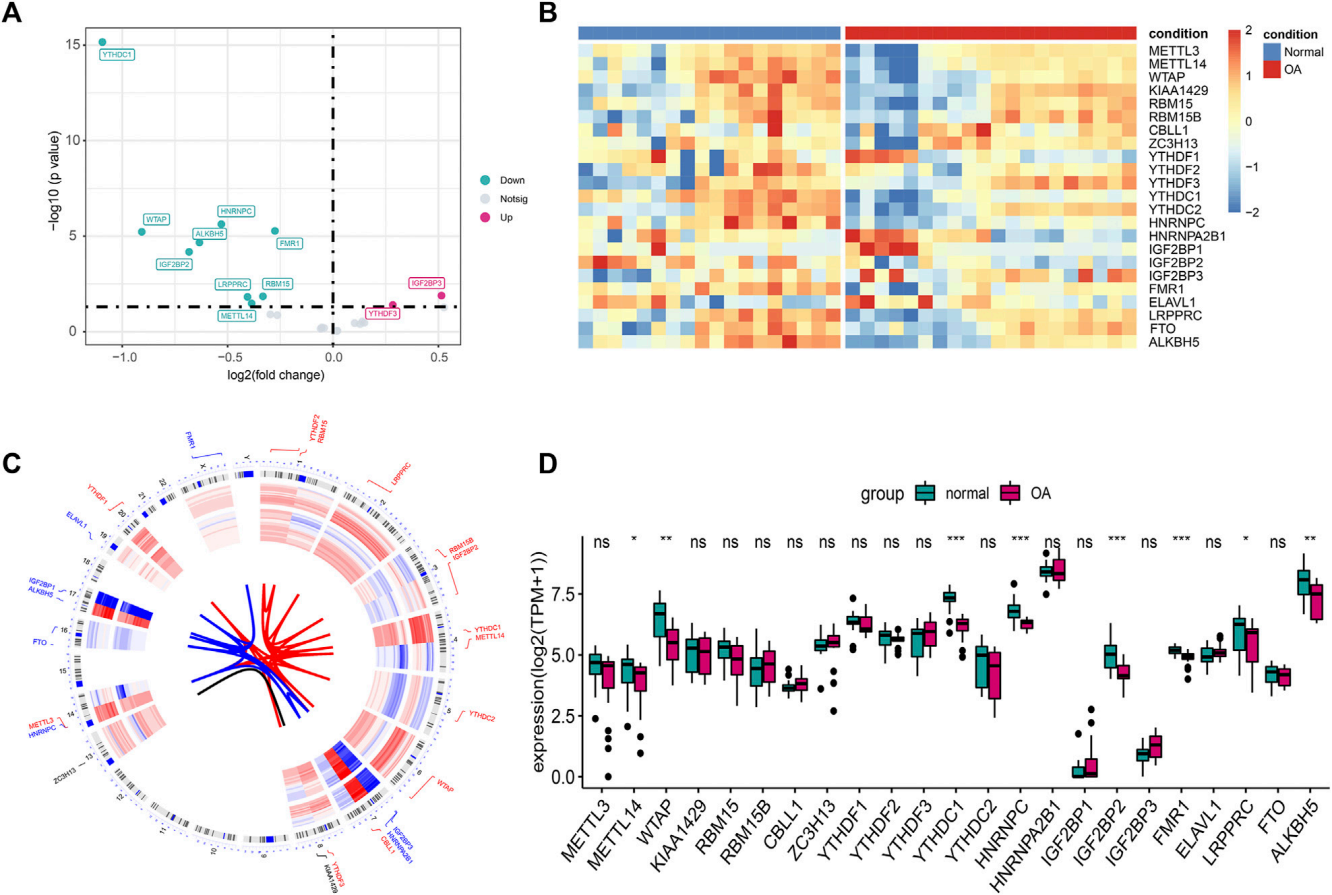
Expression differences of m6A genes in OA. **(A)** The volcano plot of m6A gene difference: the abscissa is log2FC, and the ordinate is −log10(*p*-value). **(B)** Heat map of m6A gene differences. **(C)** Chromosome circus map of differential expression of m6A gene, the inner circle is the expression heat map of OA samples, and the outer circle is the expression heat map of normal samples. **(D)** Boxplot of m6A gene differences. (ns: *p* > 0.05, *: *p* < 0.05, **: *p* < 0.01, ***: *p* < 0.001).

Figure 1 shows the flowchart associated with the analysis of the expression of m6A-related genes in OA. To analyze the effect of m6A-related gene expression values on OA tissues relative to normal tissues, differentially expressed m6A-related genes in the dataset were obtained using the DEseq2 package and the results obtained were presented as a volcano plot (Figure 2A). From this figure, it is evident that OA showed high *IGF2BP3* and *YTHDF3* expression levels; significantly low *YTHDC1* expression levels; and low *WTAP, IGF2BP2, FMR1, RBM15, ALKBH5, LRPPRC, HNRNPC*, and *METTL14* expression levels. Meanwhile, a heat map (Figure 2B), chromosomal circos map (Figure 2C), and box map (Figure 2D) were generated based on the expression of m6A-related genes. As shown in the abovementioned figures, *YTHDC1, WTAP, IGF2BP2, FMR1, ALKBH5, LRPPRC, HNRNPC*, and *METTL14* showed low expression levels in OA.

### Correlation Between the Expression Levels of the m6A-Related Genes in OA

**FIGURE 3:**
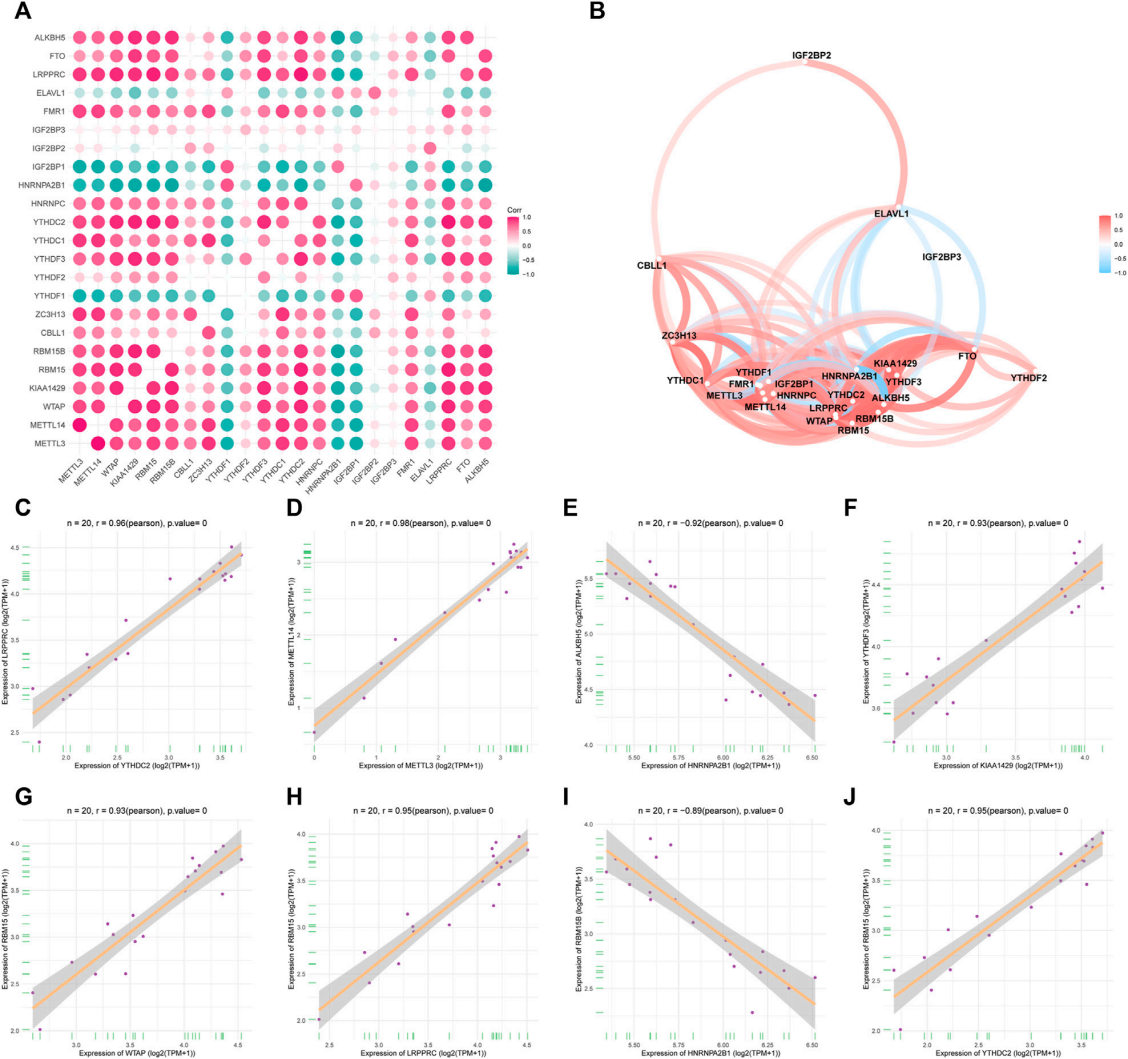
Expression correlation of m6A genes in OA. **(A)** Heatmap of 23 m6A gene expression correlations. **(B)** m6A gene expression correlation network diagram. **(C–J)** Scatter plot of some highly correlated m6A genes.

To analyze the correlations between the expression levels of the m6A-related genes in OA, we performed correlation analysis on the expression levels of the m6A-related gene. In this regard, we used the corplot packet to plot the correlation results as a heat map (Figure 3A) and network map (Figure 3B). Figures 3C–J shows the scatter diagrams corresponding to the strongly correlated m6A-related genes in OA. Our results indicated that *METTL3* expression showed a strong correlation with *METTL14* expression, *KIAA1429* expression showed a strong correlation with *YTHDF3* expression, and *RBM15* expression showed a strong correlation with *WTAP* and *LRPPRC* expression. Additionally, *HNRNPA2B1* showed a strong correlation with *RBM15B* and *ALKBH5* expression, and *YTHDC2* showed a strong correlation with *RBM15* and *LRPPRC* expression.

### Construction of an m6A Gene Prediction Model in OA

**FIGURE 4:**
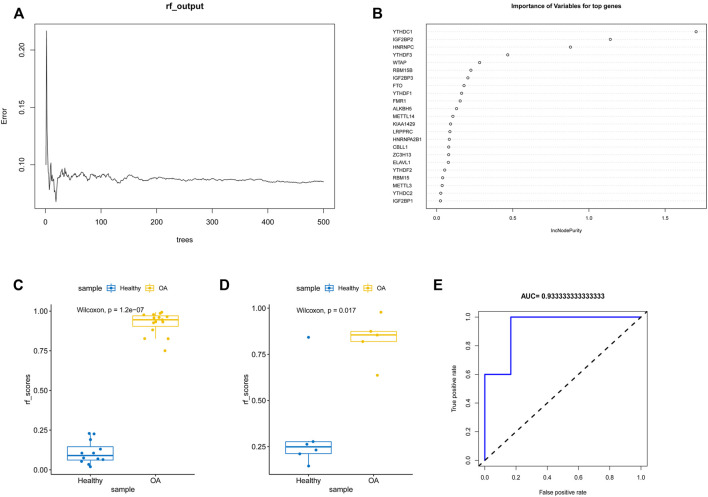
Random forest analysis. **(A,B)** The m6A gene was modeled by random forest. **(C,D)** Boxplots of ratings for training and validation sets. **(E)** ROC plot of random forest diagnosis.

**FIGURE 5:**
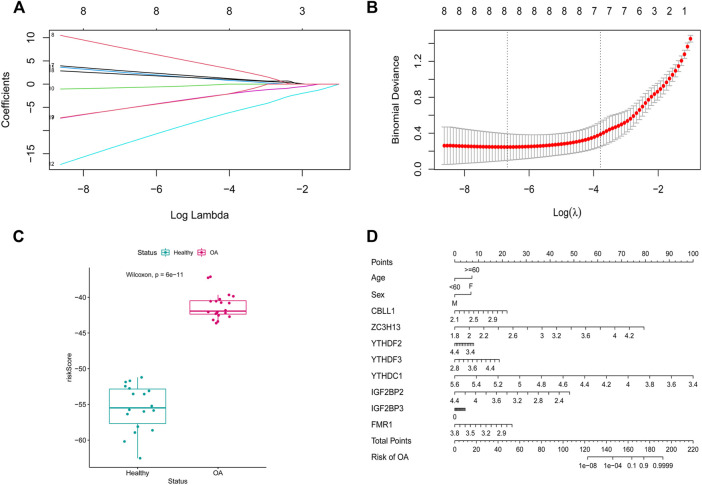
LASSO regression modeling. **(A,B)** LASSO regression modeling m6A gene. **(C)** scoring boxplot. **(D)** diagnostic Nomo plot.

To analyze the resolution of OA by m6A regulatory factors, we first used the random forest method (Figures 4A,B). Samples were randomized into a training set (70%) and a verification set (30%), and the boxplots (Figures 4C,D) obtained thereafter showed significant differences in model scores between OA and healthy groups in the training and validation sets. Further, the ROC curve (Figure 4E) showed that the model constructed using the random forest method exhibited a good diagnostic capability for OA; given that the results of the random forest analysis showed that all the m6A regulatory factors had good predictive power, we used Lasso regression analysis to characterize and reduce the m6A regulatory factors while excluding insignificant regulatory factors (Figures 5A,B). The obtained prediction model was: Risk Score = *CBLL1* * 2.731 + *ZC3H13* * 7.407 + *YTHDF2* * −0.732 + *YTHDF3* * 2.384 + *YTHDC1* *−12.361 + *IGF2BP2* * −5.030 + *IGF2BP3* * 2.051 + *FMR1* *−5.042. Furthermore, the boxplot in Figure 5C showed a significant difference in risk scores between the OA and healthy groups. Subsequently, we constructed an OA risk-related nomogram (Figure 5D), which could distinguish between healthy and OA samples as a function of risk scores.

### Correlation Between m6A Regulatory Factors in OA and Immune Cells and Immune Process

**FIGURE 6:**
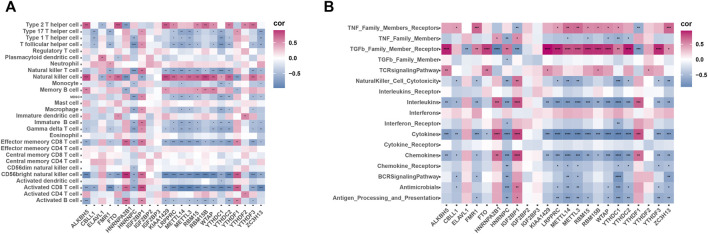
Correlation of infiltrating immune cells, immune response genes and m6A regulators. **(A)** Dot plot shows the correlation between each immune microenvironment aberrant infiltrating cell type and each abnormal m6A regulator. **(B)** Dot plot shows the correlation between each immune response genome and each M6A regulator.

To investigate the correlation between m6A regulatory factors and the immune microenvironment, we performed a correlation analysis involving m6A regulators, infiltrating immunocytes, and immune reaction gene sets (Figures 6A,B). The correlation analysis showed that m6A regulatory factors are strongly correlated with several immune cells. We also found that NK cells were positively correlated with most m6A regulatory factors, while NKT cells and CD8+T cells were negatively correlated with most m6A regulatory factors. Additionally, during the immune process, most of the m6A regulatory factors showed a negative correlation with cytokines and a positive correlation with receptors belonging to the TGFb family.

### Modification Patterns of m6A RNA Methylation Mediated by 23 Regulatory Factors in OA

**FIGURE 7:**
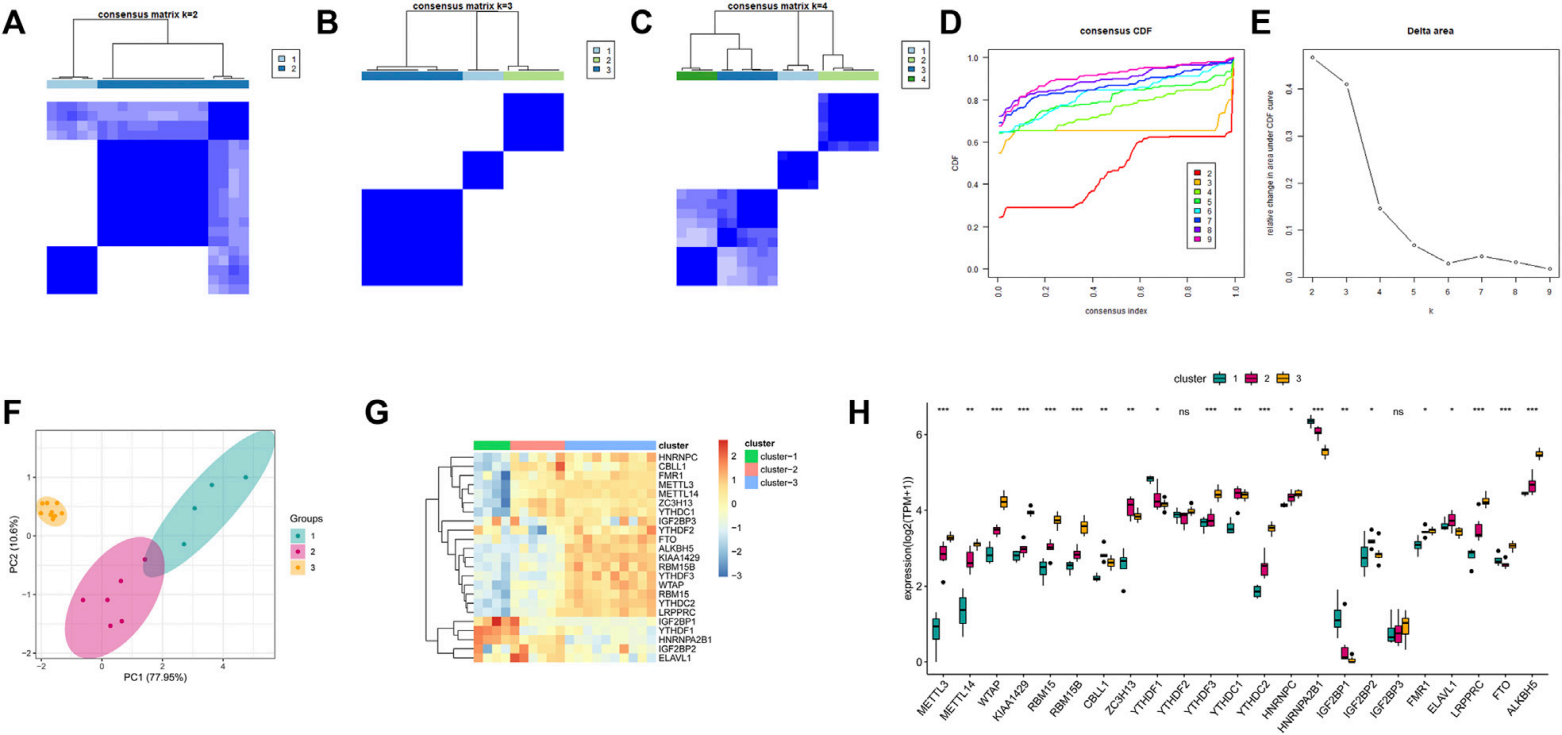
Unsupervised clustering of 23 m6A regulators. Three distinct isoforms of m6A modification patterns were identified in OA. **(A–C)** Heat map of the co-occurrence ratio matrix of OA samples when k = 2–4. **(D)** Consensus clustering cumulative distribution function when k = 2–7. **(E)** Relative area under the CDF curve when k = 2-7 Changes. **(F)** Principal component analysis of the transcriptome profiles of the 3 m6A isoforms showed that the transcriptomes of different modification patterns were significantly different. **(G)** Unsupervised aggregation of 23 m6A regulators in the 3 modification patterns Class. **(H)** Expression status of 23 m6A regulons in 3 m6A isoforms.

To investigate the m6A modification pattern of bones and joints, we performed unsupervised consensus clustering on OA samples based on the expression of 23 m6A regulatory factors (Figures 7A–E). The results thus obtained showed that clustering exhibited good stability at K = 3, and PCA revealed favorable differences among the three m6A molecular subtypes (Figure 7F). Further, the thermogram (Figure 7G) and boxplot (Figure 7H) show that the expression levels of the 23 m6A regulatory factors in the three m6A modification patterns were significantly different.

### Immune Microenvironment Characteristics of Different m6A Modification Modes

To understand the differences in the immune microenvironment characteristics of different m6A modification modes, the infiltrating immune cells, immune response genome, and *HLA* gene expression were evaluated. Our results in this regard showed different immune cells for the three patterns (Supplementary Figure S1A). Compared to Modes 2 and 3, Mode 1 showed increased immunocytes infiltration; the number of CD4+T cells, immature dendritic cells, and natural killer cells corresponding to Mode 3 were higher than those corresponding to Mode 2. Additionally, members of the TGFB receptor family showed high activity in Mode 3 (Supplementary Figure S1B), whereas the cytokine process in Mode 1 was relatively active. This was consistent with the previous analysis of the immune process. Similar trends were observed for *HLA* gene expression (Supplementary Figure S1C). In Mode 1, the overall expression of genes belonging to the *HLA* family was high. These results suggested that m6A modifications in Modes 1 and 3 mediated the master immune response, while the modification in Mode 2 mediated the mild immune response to OA. Further, the immune responses mediated by Modes 1 and 3 were different.

### Biological Characteristics of Three m6A Modification Modes

**FIGURE 8:**
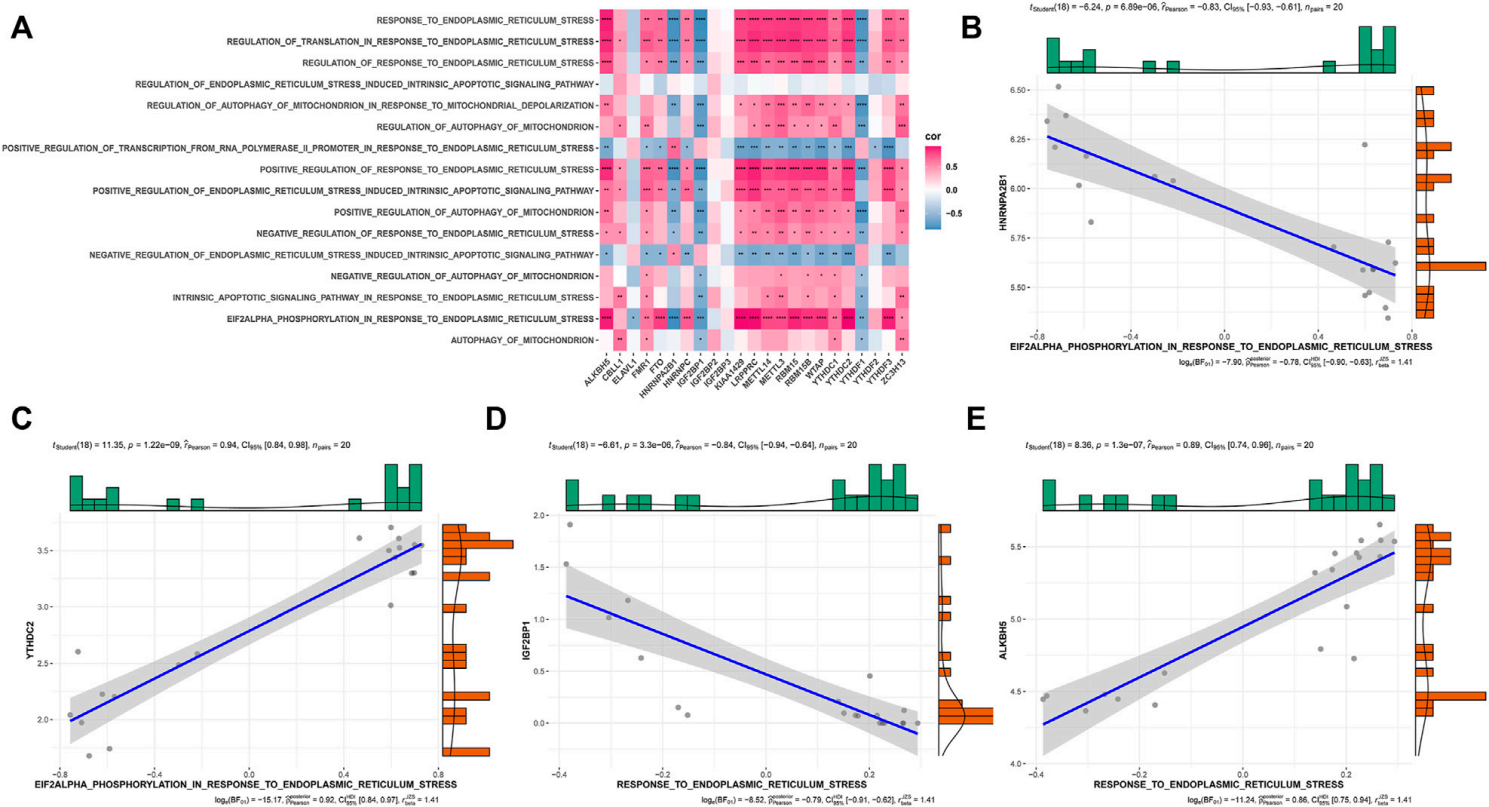
Correlation of m6A gene expression and pathways related to endoplasmic reticulum stress and mitophagy in OA. **(A)** The overall heat map of the correlation between m6A gene expression and ER stress and mitophagy-related pathways. **(B–E)** The correlation between *HNRNPA2B1, YTHDC2, IGF2BP1, and ALKBH5* gene expression and ER stress pathway.

To investigate the biological responses associated with the three m6A modification modes, we compared the HALLMARKS pathway and the GO pathway and evaluated the activation state of the biological pathway using GSVA enrichment analysis (Supplementary Figures 2A–F). Compared with Modes 1 and 3, relatively fewer pathways were enriched in Mode 2, thereby revealing the enrichment of pathways such as the ROS pathway. Meanwhile, Modes 1 and 3 were enriched in almost the same pathway. Specifically, Model 1 showed the enrichment of well-known pathways, such as the PI3K, AKT, and MTORC1 pathways, whereas Mode 3 showed the enrichment of pathways related to endoplasmic reticulum oxidative stress and mitochondrial autophagy. Previous studies have demonstrated that endoplasmic reticulum oxidative stress and mitochondrial autophagy play significant roles in OA; therefore, we investigated the correlation between m6A regulatory factors, endoplasmic reticulum oxidative stress, and mitochondrial autophagy pathway scores (Figure 8A). These results showed that the m6A regulatory factors were strongly correlated with endoplasmic reticulum oxidative stress and mitochondrial autophagy pathways (Figures 8B–E).

### m6A-Related TF-MIRNA Network Construction and Drug Development for OA Treatment

In this study, we observed high *IGF2BP3* and *YTHDF3* expression levels in OA. Based on these two genes, we constructed a transcription factor-miRNA network and a drug-compound network diagram using the Network Analyst database. As shown in Supplementary Figure S3A, formaldehyde, C646, and other compounds can simultaneously act on these two regulatory factors. This observation suggests that formaldehyde and C646 compounds have the potential to be used as therapeutic drugs. As shown in Supplementary Figure S3B, *SREBF1, SREBF2*, and *EGR1* were identified as the common targeted transcription factors of the two genes, while hsa-miR-590-3p and hsa-miR-340 were identified as the commonly targeted miRNAs of the two genes.

### RT-qPCR and Western Blotting

**FIGURE 9:**
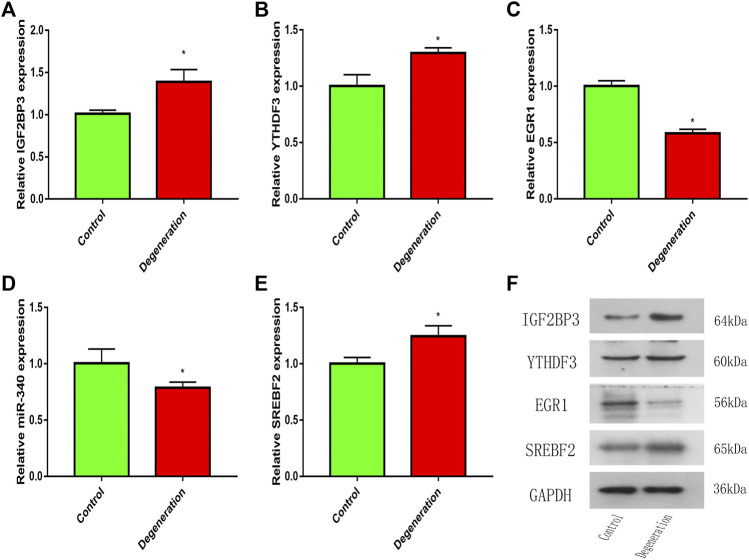
The mRNA or miRNA expression in the control and degeneration group. **(A,B)** Expression level of m6A genes in the control and degeneration group. **(C–E)** Expression level of m6A-related transcription factor and miRNA in the control and degeneration group. **(F)** Expression level of m6A protein and m6A-related transcription factor in the control and degeneration group. *p < 0.05 vs. Control.

The expression levels of IGF2BP3 and YTHDF3 were upregulated in the IL-1β-induced degeneration group (Figures 9A,B). This is consistent with the results of the microarray analysis. Further, RT-qPCR results showed the expression of EGR1 and miR-340 of degeneration group was significantly decreased, while the expression of SREBF2 of degeneration group was upregulated (Figures 9C–E). These qPCR results for the two groups showed statistically significant differences. On the other hand, we also detected the protein expression levels of IGF2BP3, YTHDF3, EGR1, and SREBF2 by western blotting, and the results were consistent with PCR, IGF2BP3, YTHDF3, and SREBF2 were upregulated in the IL-1β-induced degeneration group, and the expression of EGR1 of degeneration group was significantly decreased. (Figure 9F).

## Discussion

OA is a disease that involves biomechanics, inflammation, and complex biological responses of the immune system (Woodell May and Sommerfeld, 2019). Presently, there are still many research gaps concerning the immune regulatory mechanism of OA, especially m6A regulatory factor-mediated immune regulation. In this study, we systematically investigated the modification pattern of m6A in the OA immune microenvironment; based on the results obtained, we arrived at the following conclusions:

First, we observed a difference in the expression of some m6A regulatory factors between healthy and OA samples, specifically the upregulation of *IGF2BP3* and *YTHDF3* in OA samples. Consistent results were obtained via RT-qPCR and WB, which validated our findings. These results indicated that m6A regulatory factors, especially *IGF2BP3* and *YTHDF3*, may be involved in OA development. Additionally, we observed the existence of correlations between the expression levels of m6A regulatory factors. Thus, we speculated that in OA, m6A regulatory factors jointly regulate OA progression through a regulatory network.

Next, we established an m6A classifier using m6A regulators that offer the possibility to distinguish between healthy and OA samples. This reaffirmed the important role of m6A regulatory factors in OA. Further, to improve and more conveniently predict OA, we established a new m6A nomogram. Based on the review of relevant literature, we found that nomograms are rarely used to predict OA occurrence and progression. Daniel et al. developed a nomogram for diagnosing the rapid progression of knee osteoarthritis. Similarly, we established an m6A nomogram for predicting the risk of OA from the perspective of m6A modifications (Riddle et al., 2016). Different scores were assigned to factors such as age, sex, *CBLL1, ZC3H13, YTHDF2, YTHDF3, YTHDC1, IGF1BP2, IGF2BP3*, and *FMR1*. The total score was obtained by adding the scores of each factor. The total score was less than 160, the probability of OA was less than 0.1, and probability of OA was greater than 0.9 if the total score was greater than 180.

Moreover, we investigated the association between m6A regulatory factors and the immune properties of OA, including the gene set for immune cell infiltration and immune response. We found that many m6A regulatory factors are closely related to these immune characteristics. Unsupervised clustering of periodontitis samples using m6A regulator expression profiles led to three subtypes with distinctive m6A modification patterns, and each subtype exhibited unique immune characteristics. Considering that each subtype has its immune characteristics, we believe that classification based on immunophenotypes of different m6A modulators is feasible. We believe that this classification strategy for immune subtypes will help in comprehensively understanding the mechanisms of immune regulation.

Notably, *YTHDF3* was upregulated in OA samples. In an immune correlation study, *YTHDF3* was positively correlated with type II T helper cells and TGFb family member receptors. In Mode 3, the expression of *YTHDF3*, type II T helper cells, and TGFb family member receptors were upregulated. Mode 3 could be enriched in pathways related to endoplasmic reticulum oxidative stress and mitochondrial autophagy. Meanwhile, we found that the m6A regulatory factor also had a strong correlation with endoplasmic reticulum oxidative stress and mitochondrial autophagy pathway, which is consistent with our previous study. Small extracellular vesicles-miR-151a-3p targeted *YTHDF3* to reduce the transcriptional inhibitory activity of *SP3*, promote the transactivation of TGF-β1 in KCs, and then activate the *SMAD2/3* pathway to enhance the stem cell-like characteristics of the incoming GC cells (Li et al., 2021). TGF-β 1 plays a vital role in maintaining the homeostasis of articular cartilage and subchondral bone (He et al., 2020). Apoptosis of articular chondrocytes is related to ROS-induced oxidative stress, which leads to mitochondrial damage and activates endoplasmic reticulum stress (Feng et al., 2019). Mitochondrial autophagy disorders in chondrocytes accelerate the development of OA (Wang et al., 2020). This suggests that *YTHDF3*, type II helper cells, TGFb family member receptors, endoplasmic reticulum oxidative stress, and mitochondrial autophagy pathways are closely related to OA.

Furthermore, we established a transcription factor-miRNA-m6A regulatory factor network and a drug-compound-m6A regulatory factor network. *SREBF1*, *SREBF2*, and *EGR1* are common transcription factors of *IGF2BP3* and *YTHDF3*, while hsa-miR-590-3p and hsa-miR-340 are two miRNAs that can be combined by both. Compounds such as formaldehyde and *C646* can be combined with these two regulatory factors simultaneously.

*SREBP-1* is a transcription factor responsible for the expression of enzymes involved in lipid and cholesterol homeostasis under sterol stimulation. The adsorption of glucose on chitosan membranes (CTS-Glc) stimulated the proliferation of human chondrocytes by providing energy through the regulation of lipogenesis via SREBP-1/Fans and promoting the cell cycle process through the expression of cell cycle regulators induced by *SREBP-1* (Chang et al., 2017).

The cholesterol regulatory element-binding factor-2 (*SREBF2*) gene is a well-known transcriptional regulator of the cholesterol biosynthesis genes. Stigmasterol (*STM*) reduces IL-1β-induced *ATDC5* cell injury in mouse chondrocytes via *SREBF2*, and *STM* reduces il -1β-induced *ATDC5* cell iron ptosis via *SREBF2* (Mo et al., 2021). *SIRT1* may aggravate osteoarthritis cartilage degeneration by activating the *SREBP2* protein-mediated PI3K/AKT signaling pathway (Yu et al., 2016).

*EGR1* (early growth response 1) is a transcription factor of the c2h2 type zinc finger protein EGR family that regulates chondrocyte hypertrophy by activating the β-catenin signaling pathway (Sun et al., 2019). The *EGR1* gene has been identified as the central gene of OA development (Chen et al., 2021). *EGR1*, cartilage degeneration, and the expression of *EGR1* in the articular cartilage of OA patients increased (Huan et al., 2021). Mir-340-5p may inhibit the ERK signaling pathway through the *FMOD* gene, promote the proliferation of OA mouse chondrocytes, and inhibit apoptosis (Zhang et al., 2019).

To examine the expression of these genes in healthy and IL-1b-induced osteoarthritis samples, we performed RT-qPCR and confirmed that *IGF2BP3* and *YTHDF3* were closely associated with the development of OA and *SREBF2, EGR1*, and miR-340 could be involved in OA progression by regulating the expression of I*GF2BP3* and *YTHDF3*. However, this study had some limitations. First, the data were downloaded from a public database and could not be evaluated for input errors. Second, based on bioinformatics analysis, RT-qPCR was used to detect the difference in molecular expression between OA and healthy samples; However, flow cytometry still need to be supplemented to verify the role of the molecules and the potential mechanism of OA. Single cells can also be sequenced to obtain the most accurate number of immune cells.

In conclusion, our study revealed a potential regulatory mechanism of m6A modification in the immune microenvironment of OA. Different modification modes of m6A cannot be ignored as they affect the immune microenvironment of OA, thus influencing the occurrence and development of OA. A comprehensive analysis of the modification mode of OA m6A in our study will help to understand the immune regulatory mechanism of OA, provide a reference for the treatment of OA, and supplement the research blank in this field. Meanwhile, the developed m6A OA nomogram can help assess the risk of OA, thus providing a reference for the clinical diagnosis of OA.

## Data Availability

The datasets presented in this study can be found in online repositories. The names of the repository/repositories and accession number(s) can be found in the article/Supplementary Material.

## Supplementary Material

Supplementary Figure S1 Differences in immune microenvironment characteristics between different m6A modification patterns. **(A)** Differences in the abundance of infiltrating immune cells in each immune microenvironment under the three M6A modification patterns. **(B)** differences in the activity of each immune response genome under the three M6A modification patterns. **(C)** HLA genes in the three m6A modifications Expression differences in patterns.

Supplementary Figure S2 There are differences in the underlying biological functional characteristics of the three m6A modification patterns. **(A,B)** The difference between the enrichment integrals of M6A modification mode 1 and mode 2 signaling pathways. **(B)** the difference between the enrichment integrals of M6A modification mode 1 and mode 3 signaling pathways. **(C)** M6A modification mode 2 and mode 3 signaling pathways Differences in enrichment scores.

Supplementary Figure S3 Network graph analysis of highly expressed m6A genes in OA. **(A)** The interaction network of *YTHDF3* and *IGF2BP3* with compounds. **(B)** The transcription factor and miRNA binding network of YTHDF3 and IGF2BP3.
