# Supplementary figures and images for: m6A Regulator-Mediated RNA Methylation Modification Patterns Regulate the Immune Microenvironment in Osteoarthritis
Source: Front Genet. 2022 Jun 23;13:921256. doi: 10.3389/fgene.2022.921256 (PMC9262323; doi:10.3389/fgene.2022.921256)

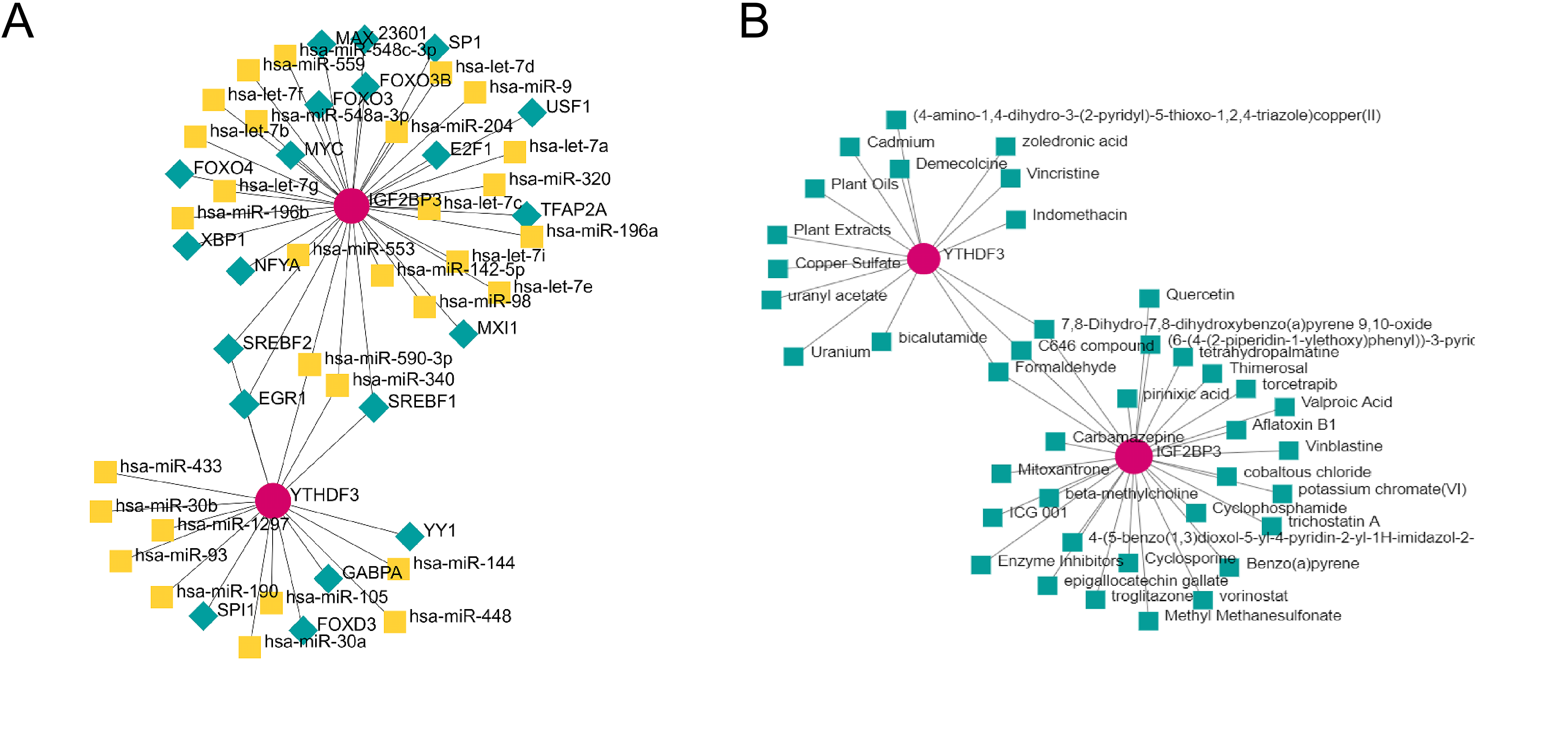

Supplement: Supplementary file 1 [file Image3.TIFF]

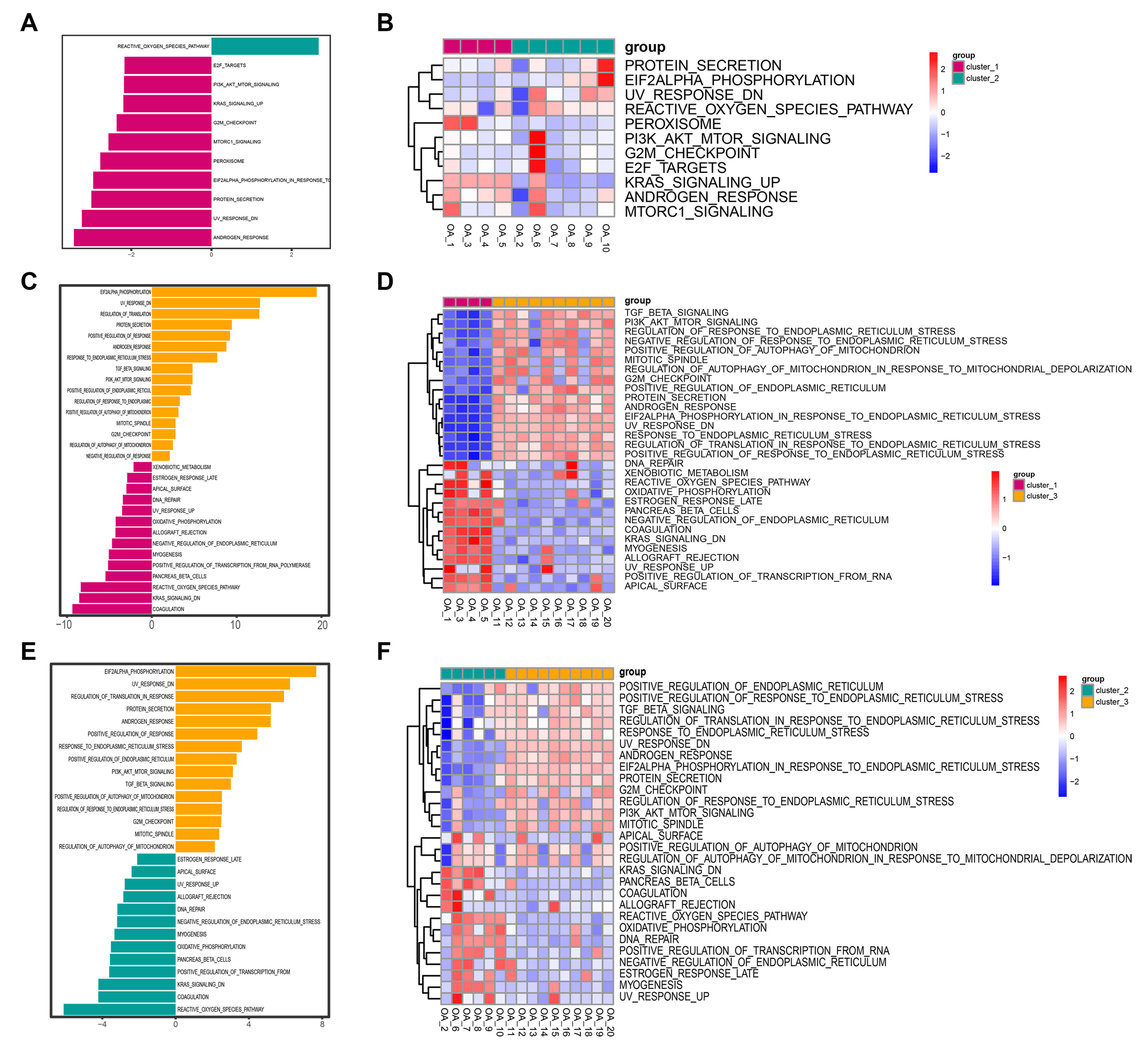

Supplement: Supplementary file 3 [file Image2.TIF]

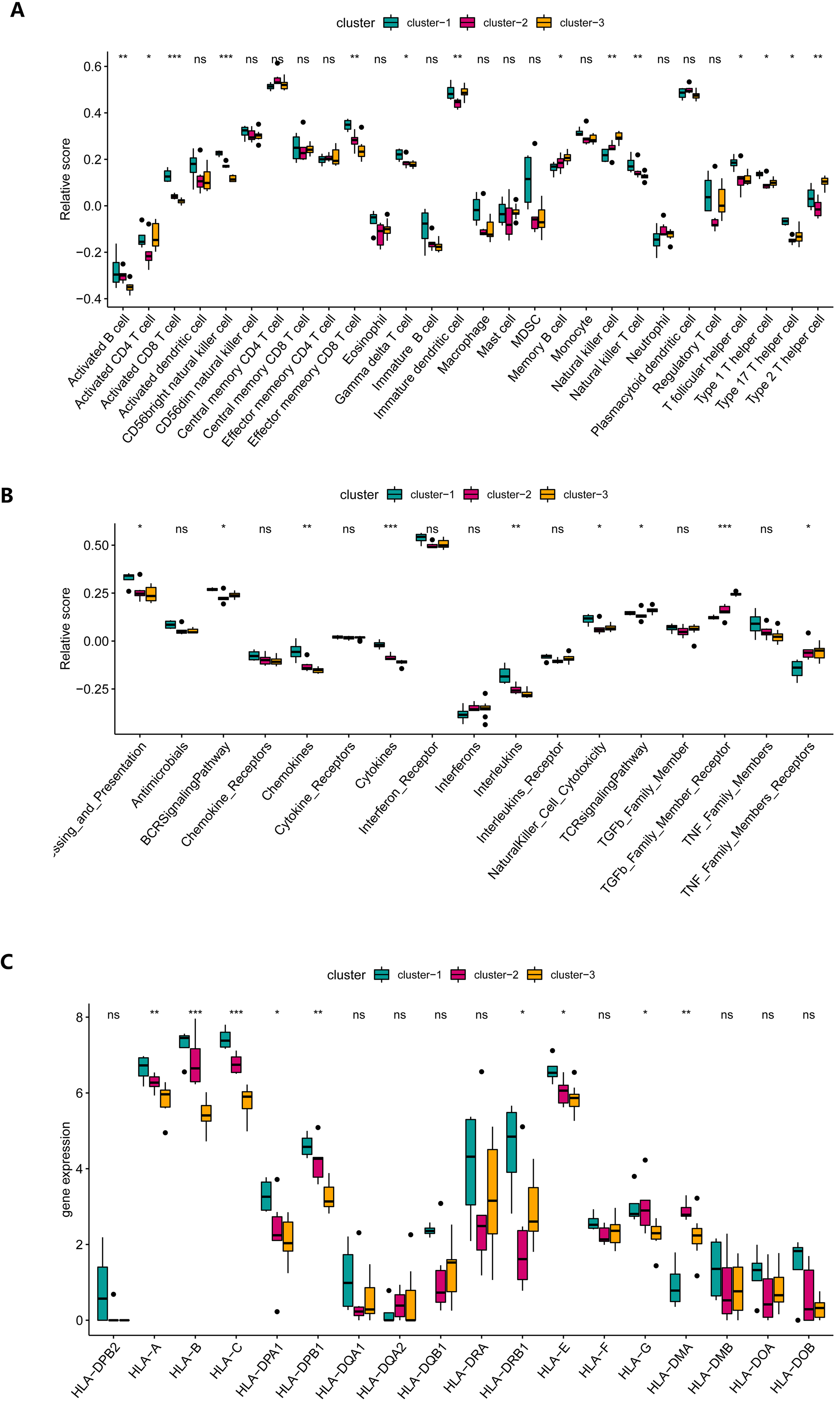

Supplement: Supplementary file 4 [file Image1.TIF]
