# Supplementary material for: m6A Regulator-Mediated RNA Methylation Modification Patterns Regulate the Immune Microenvironment in Osteoarthritis
Source: Front Genet. 2022 Jun 23;13:921256. doi: 10.3389/fgene.2022.921256 (PMC9262323; doi:10.3389/fgene.2022.921256)
Supplement: Supplementary file 2 [file Table1.DOCX]

| Gene | Forward primer (5’-3’) | Reverse primer (5’-3’) |
| --- | --- | --- |
| IGF2BP3 | GGACCACCAGAGGCTCAGTTCA | CCAGCAGCAAAGGATGGCACTC |
| YTHDF3 | GCCTCAACCACCACAACCACAG | CACTGCCCGCTCCATTGTTCTG |
| SREBF2 | ACGCCAACATTCAGCACCACTC | TGGGAGGATGTCACCAGGCTTT |
| EGR1 | CCAACAGTGGCAACACCTTG | GTGGGTTGGTCATGCTCACT |
| GAPDH | AAGTATGACAACAGCCTCAAG | TCCACGATACCAAAGTTGTC |
| miR-340 | GTCGTATCCAGTGCGTGTCGTGGAGTCG | GCAATTGCACTGGATACGACAATCAG |
| U6 | CTCGCTTCGGCAGCACATATACT | ACGCTTCACGAATTTGCGTGTC |

TABLE S1. mRNA and miRNA primer sequences
